# P-1451. Impact of Simulation-based communication training on addressing pediatric vaccine hesitancy in Japan

**DOI:** 10.1093/ofid/ofaf695.1637

**Published:** 2026-01-11

**Authors:** Tatsuki Ikuse, Akihiko Saitoh, Samreen Vora, Sarah Schaffer DeRoo, Brande Brown, Janna Patterson, Hannah Foehringer Merchant, Kathryn E Lander, Mary Claire Eyre, Satoshi Kamidani

**Affiliations:** National center for child health and development, Japan Pediatric Society, Setagaya, Tokyo, Japan; Niigata University, Niigata, Niigata, Japan; American Academy of Pediatrics, Itasca, Illinois; American Academy of Pediatrics, Itasca, Illinois; American Academy of Pediatrics, Itasca, Illinois; American Academy of Pediatrics, Itasca, Illinois; American Academy of Pediatrics, Itasca, Illinois; American Academy of Pediatrics, Itasca, Illinois; American Academy of Pediatrics, Itasca, Illinois; American Academy of Pediatrics, Itasca, Illinois

## Abstract

**Background:**

Vaccine hesitancy continues to pose a significant barrier to pediatric vaccine uptake globally. Although growing evidence supports the use of presumptive approach and motivational interviewing (MI) as effective strategies to address vaccine hesitancy, data on their implementation and impact outside the United States remains limited. This study evaluated the impact of a simulation-based communication training for pediatric physicians in Japan on their perceptions and confidence in addressing vaccine hesitancy.

**Fig 1.**
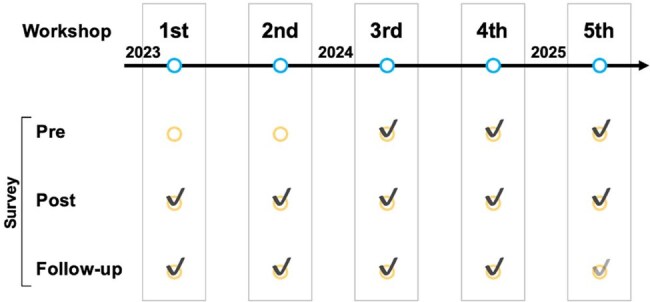
Held workshops and pre, post, and follow-up survey for participants in Japan Five workshops were held in Japan from January 2023 to April 2025. The status of surveying workshop participants is indicated by a check mark (Follow-up surveys for the fifth workshop are ongoing and not included in this analysis). Pre-survey was started from the third workshop.

**Table 1.**
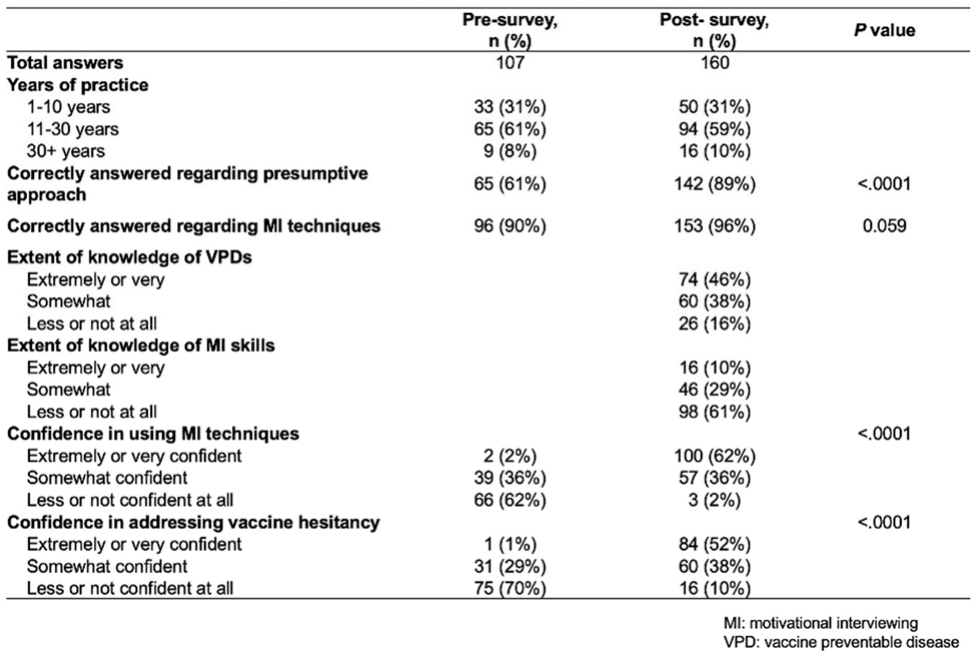
Results of pre- and post-survey of the simulated training of vaccine hesitancy* *Survey responses were analyzed as aggregated data.

**Methods:**

From January 2023 to April 2025, five simulation-based workshops were conducted to train pediatric vaccinators in presumptive approach and MI. Participants received pre-recorded lectures on vaccine hesitancy and communication strategies prior to attending. Surveys were administered at 3 timepoints: pre-intervention (post-lecture), immediately post-workshop, and follow-up ( >3 months post-workshop).

**Table 2.**
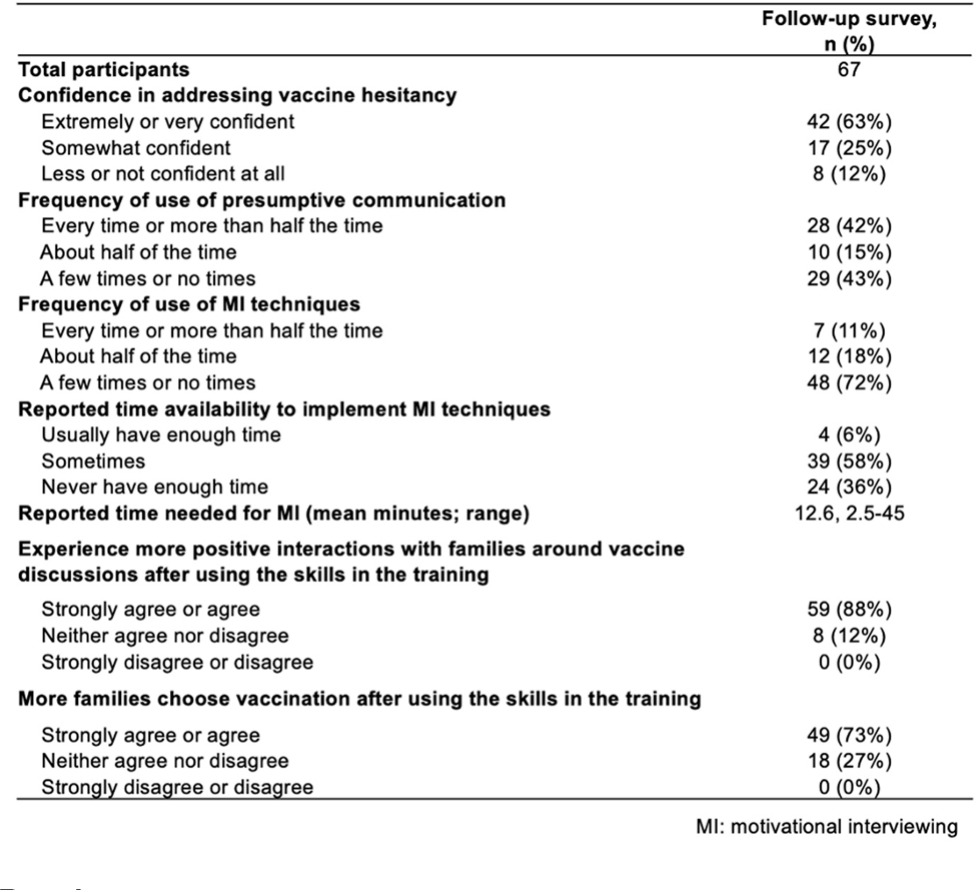
Results of follow-up survey of the simulated training of vaccine hesitancy

**Results:**

A total of 178 physicians participated in the workshops (Fig. 1). In total, 107 pre-surveys and 160 post-surveys were completed and the majority of participants (61%) had 11–30 years of clinical experience (Table 1). While 84% had baseline knowledge of vaccine-preventable diseases, 61% reported limited familiarity with MI techniques. Prior to the workshops, only 1% of physicians expressed high confidence in addressing vaccine hesitancy; this significantly increased to 52% post-workshop (*p* < .0001). Follow-up data showed that 63% sustained high confidence (Table 2). While 57% reported frequent use (≥50% of the time) of presumptive approach, only 29% reported frequent use of MI and 36% reported inadequate time to implement MI. Furthermore, 88% reported more positive interactions with families regarding vaccines, and 73% observed increased parental acceptance of recommended vaccines.

**Conclusion:**

Simulation-based training in presumptive approach and MI techniques significantly improved and sustained physicians’ confidence in addressing pediatric vaccine hesitancy in Japan, with perceived impacts on clinical interactions and vaccine acceptance. Further evaluation of training implementation in other countries and assessment of caregiver perceptions are ongoing.

**Disclosures:**

Sarah Schaffer DeRoo, MD, MA, FAAP, Vindico Medical Education: Advisor/Consultant Satoshi Kamidani, MD, PhD, FAAP, Bavarian Nordic: Grant/Research Support|Meissa: Grant/Research Support|Moderna: Grant/Research Support|Pfizer: Grant/Research Support|Sanofi: Grant/Research Support

